# Supplementary material for: Charge storage in oxygen deficient phases of TiO2: defect Physics without defects
Source: Sci Rep. 2016 Jul 1;6:28871. doi: 10.1038/srep28871 (PMC4929474; doi:10.1038/srep28871)
Supplement: Supplementary Information [file srep28871-s1.pdf]

Charge storage in oxygen deficient phases of TiO_2: defect Physics without defects.

Padilha, A. C. M.[1,2,*], Raebiger, H.[1,2], Rocha, A. R.[3], and Dalpian, G. M.[1,+]

[1]Centro de Ciências Naturais e Humanas, Universidade Federal do ABC, Santo André, SP, Brazil
[2]Department of Physics, Yokohama National University, Yokohama, Japan
[3]Instituto de Física Teórica, Universidade Estadual Paulista, São Paulo, SP, Brazil
[*]antonio.padilha@ufabc.edu.br
[+]gustavo.dalpian@ufabc.edu.br

SUPPLEMENTARY MATERIAL

Bellow we present Supplementary data for the main article. As noted in the main manuscript for the oxygen deficient phases of TiO_2, the shear planes lead to the formation of an intermediate filled band that lies in what would be the gap of defect free TiO_2. In figure Supplementary Figure S1 we present a schematic plot of the intermediate band for the materials studied. From the figure one can see that the intermediate band (IB) when discharged is promoted to higher energies inside the conduction band (CB) of the host material (TiO_2-rutile) while the valence band (VB) remains unchanged. As the system can go from the neutral charge state to the $+4$ charge state, each unit cell can accommodate up to $+4e$ of charge.

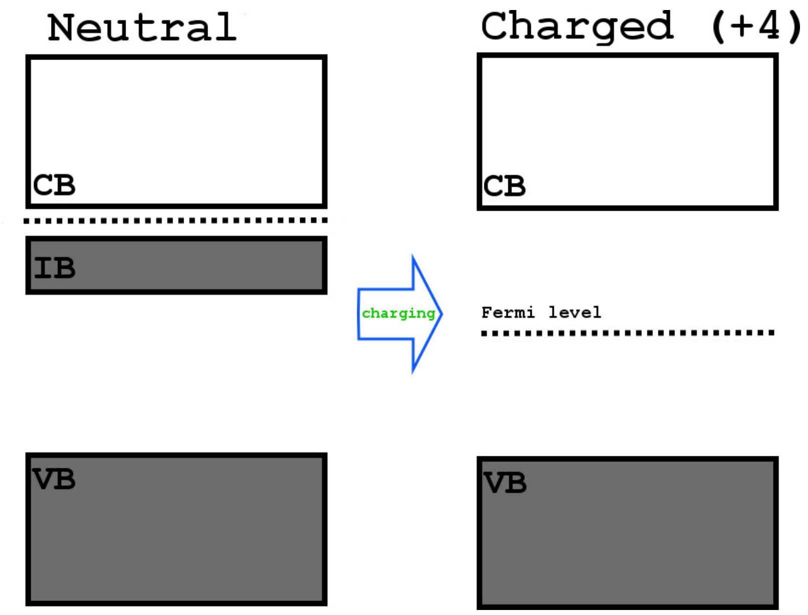

Figure Supplementary Figure S1. (color online) Schematic diagram of the energy storage process on the oxygen-deficient compounds presented in this work: Ti_2O_3, Ti_4O_7, and Ti_5O_9.

In the main manuscript the unit cell was kept fixed as recent experiments have shown evidence that oxygen-deficient phases might be obtained within a TiO_2-rutile matrix.[1,2] At the same time, we have shown in the main manuscript that it is possible to obtain the Magnéli phases by performing transformations that leave the supercell unchanged. This was also addressed in a previous work in our group where we have calculated the band alignment between TiO_2-rutile and one of the Magnéli phases. In that case we showed that one obtains a type-II band alignment, which could mean that one could use the TiO_2 to charge/decharge the material, *i.e.* one could build a charge storage device entirely from the same material (electrode + active region).

Table Supplementary Table S1. Calculated lattice parameters after structural relaxation of Ti_2O_3, Ti_4O_7, and Ti_5O_9 for different charge states representing the charging process of those compounds.

			Ti_2O_3			
q (|e|)	a(Å)	b(Å)	c(Å)	α(°)	β(°)	γ(°)
0	5.519	-	-	55.11	-	-
1	5.297	-	-	55.99	-	-
2	5.126	-	-	57.03	-	-
3	4.910	-	-	59.21	-	-
4	4.708	-	-	61.37	-	-
			Ti_4O_7			
q (|e|)	a(Å)	b(Å)	c(Å)	α(°)	β(°)	γ(°)
0	5.655	6.930	7.072	63.85	108.41	103.85
1	5.573	6.839	6.968	63.93	108.23	103.71
2	5.496	6.707	6.859	63.94	108.01	103.79
3	5.683	6.756	6.616	55.38	110.80	106.58
4	5.682	6.638	6.588	54.03	112.46	107.01
			Ti_5O_9			
q (|e|)	a(Å)	b(Å)	c(Å)	α(°)	β(°)	γ(°)
0	5.606	7.100	8.871	97.08	112.44	108.19
1	5.548	7.010	8.770	97.15	112.40	107.93
2	5.494	6.924	8.650	97.11	112.50	107.73
3	5.444	6.834	8.531	97.00	112.64	107.52
4	5.448	6.723	8.304	95.14	113.50	108.04

For the neutral case, our lattice parameters are in excellent agreement with experimental measurements of Le Page and Marezio,[3] taking in consideration that we used the primitive unit cell while Le Page used a larger one. Nevertheless, structural relaxation was performed to asses the change in volume of the systems. The unit cell parameters for all the charge states of the relaxed structures are presented in table Supplementary Table S1. The volume variation as the system is charged is relatively large for Ti_2O_3 but in the other cases, for Ti_4O_7 and Ti_5O_9 the structural relaxation is much smaller.

Using the structural parameters in Table Supplementary Table S1 we performed calculations to obtain the formation enthalpy curves. Those were calculated following the same procedure described in the main manuscript except for the unit cell size. The results are presented in figure Supplementary Figure S2. Similarly to the fixed-cell case, the charged state $+4$ is the most stable for a significant interval of occupations close to the valence band maximum (VBM). Relaxation leads to the lowering in energy of the $+2$ curve, thus revealing the transitions $\varepsilon(4^+/2^+)$ and $\varepsilon(2^+/0)$. The main results from the manuscript are qualitatively similar, namely the negative-U behavior and the fact that the system can store charge for a wide range of energies.

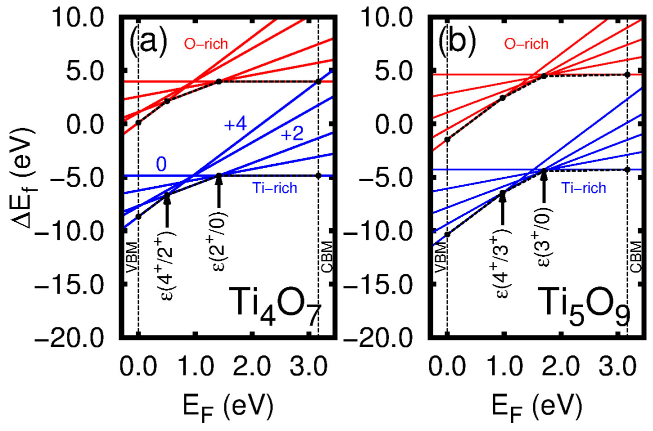

Figure Supplementary Figure S2. Formation enthalpies for all charge states of (a) Ti_4O_7, and (b) Ti_5O_9 when the cell is allowed to relax to an energy minimum. The thick black lines emphasize the lowest energy charge state for each occupation for the entire band gap span, and the charge transitions are featured as $\varepsilon(4^+/2^+)$, $\varepsilon(4^+/3^+)$, $\varepsilon(3^+/0)$, and $\varepsilon(2^+/0)$.

The effect of removing electrons from Ti_4O_7 is the spread and increase in energy of the IB, as shown by DOS plots in figure Supplementary Figure S3. As the only charge state where the Fermi energy is lower than neutral is +4, the system will suffer a quadrupole transition $\varepsilon(+4/0)$ and store positive charge. A similar effect is observed for Ti_5O_9 and is presented as figure 4 in the main manuscript.

Finally we also compared the DOS considering the relaxed system for different charge states. Both situations where the unit cell is fixed or allowed to relax are presented for comparison purposes in figure Supplementary Figure S3. It is evident that the relaxation does not affect the charging process even though the volume of the unit cell becomes smaller when charged (see table Supplementary Table S1).

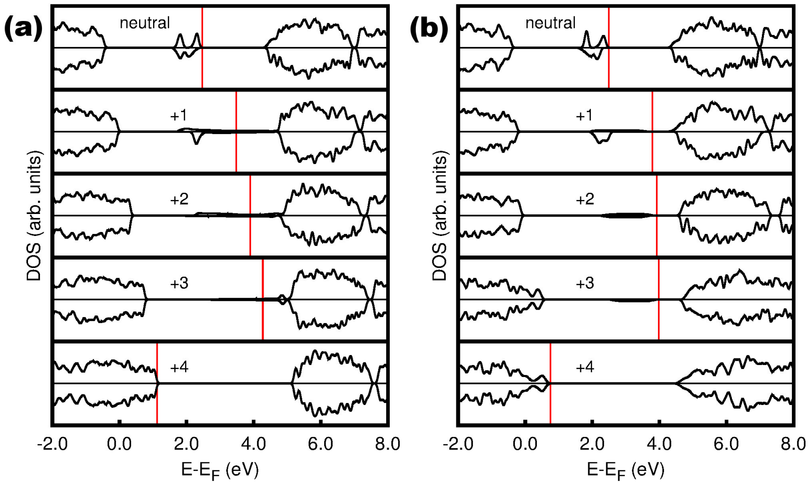

Figure Supplementary Figure S3. Density of States (DOS) for all charge states of Ti_4O_7 when the cell is fixed (a), and when it is allowed to relax to an energy minimum (b). The two spin components are represented by positive and negative values along vertical axis. The vertical full red line indicates the last occupied electronic level. Energies are referenced from the last occupied level of the host material (TiO_2-rutile)

References

1. Kwon, D.-H. *et al.* Atomic structure of conducting nanofilaments in TiO_2 resistive switching memory. *Nature Nanotech.* **5**, 148 – 153 (2010).

2. Hwan Kim, G. *et al.* Improved endurance of resistive switching TiO_2 thin film by hourglass shaped Magneli filaments. *Appl. Phys. Lett.* **98**, 262901 (2011).

3. Le Page, Y. & Marezio, M. Structural chemistry of Magnéli phases $Ti_nO_{2n-1}(4 \leq n \leq 9)$: IV. superstructure in Ti_4O_7 at 140 K. *J. Sol. State Chem.* **53**, 13–21 (1984).
